# Deficits in executive functions among youths with autism spectrum disorders: an age-stratified analysis

**DOI:** 10.1017/S0033291715002238

**Published:** 2016-03-21

**Authors:** S.-F. Chen, Y.-L. Chien, C.-T. Wu, C.-Y. Shang, Y.-Y. Wu, S. S. Gau

**Affiliations:** 1 Department of Psychiatry, National Taiwan University Hospital & College of Medicine, Taipei, Taiwan; 2 Department of Psychiatry, Taipei Tzu Chi General Hospital, Buddhist Tzu Chi Medical Foundation, Taipei, Taiwan; 3 School of Occupational Therapy, College of Medicine, National Taiwan University, Taipei, Taiwan; 4 Graduate Institute of Clinical Medicine, College of Medicine, National Taiwan University, Taipei, Taiwan; 5 Department of Psychiatry, Chang Gung Memorial Hospital-Linkou, Taoyuan, Taiwan; 6 Department of Psychology, Graduate Institute of Brain and Mind Sciences, Graduate Institute of Epidemiology and Preventive Medicine, National Taiwan University, Taipei, Taiwan

**Keywords:** Age effect, autism spectrum disorders, executive functions

## Abstract

**Background:**

Impaired executive function (EF) is suggested to be one of the core features in individuals with autism spectrum disorders (ASD); however, little is known about whether the extent of worse EF in ASD than typically developing (TD) controls is age-dependent. We used age-stratified analysis to reveal this issue.

**Method:**

We assessed 111 youths with ASD (aged 12.5 ± 2.8 years, male 94.6%) and 114 age-, and sex-matched TD controls with Digit Span and four EF tasks of the Cambridge Neuropsychological Test Automated Battery (CANTAB): Spatial Span (SSP), Spatial Working Memory (SWM), Stockings of Cambridge (SOC), and Intradimensional/Extradimensional Shift Test (I/ED).

**Results:**

Compared to TD controls, youths with ASD performed poorer on the Digit Span, SWM, SOC, and I/ED tasks. The performance of all the tasks improved with age for both groups. Age-stratified analyses were conducted due to significant age × group interactions in visuospatial planning (SOC) and set-shifting (I/ED) and showed that poorer performance on these two tasks in ASD than TD controls was found only in the child (aged 8–12 years) rather than the adolescent (aged 13–18 years) group. By contrast, youths with ASD had impaired working memory, regardless of age. The increased magnitude of group difference in visuospatial planning (SOC) with increased task demands differed between the two age groups but no age moderating effect on spatial working memory.

**Conclusions:**

Our findings support deficits in visuospatial working memory and planning in youths with ASD; however, worse performance in set-shifting may only be demonstrated in children with ASD.

## Introduction

Autism spectrum disorder (ASD) is a complex neurodevelopmental disorder with long-lasting neurocognitive dysfunctions in addition to impaired socio-communication and restricted, repetitive and stereotypical patterns of behavior, interests, and activities (APA, 2013). The prevalence of ASD has increased in recent decades causing much health burden (Matson & Kozlowski, 2011; Baxter *et al.* 2015). In Western society, the prevalence of ASD rose from 0.30% (Martin *et al*. 2007) to 2.00% (Blumberg *et al.* 2013). The increased prevalence and incidence of ASD was also evidenced in Taiwan where the number of identified ASD individuals aged 3–17 years increased from 3995 to 8072 from 2004 to 2010, and the prevalence of all age groups increased over years (Lai *et al.* 2012, 2013; Lin, 2015). These convergent evidences (Nakahachi *et al.* 1994; Gabig, 2008; Robinson *et al.* 2009) strongly suggest that ASD as a common disorder warrants much more studies to investigate the inherent deficits of ASD beyond the core symptoms.

Among the three main cognitive theories of ASD [i.e. theory of mind (Baron-Cohen *et al.* 2000), weak central coherence (Baron-Cohen, 2002), and executive dysfunction (Hill, 2004)], Hill's theory of executive dysfunction explains deficits in initiating new non-routine actions and links the behavioral repetition and perseveration to frontal lobe dysfunction in ASD. Such a theory is supported by several early studies (Damasio & Maurer, 1978; Osterling & Dawson, 1994; Dawson *et al.* 1998; Salmond *et al.* 2003); for example, the lack of joint attention in young children with ASD is related to dysfunction of the ventromedial prefrontal cortex (Osterling & Dawson, 1994). Damasio & Maurer (1978) were the first to report that individuals with ASD, similar to patients with frontal lobe damage, had difficulties in switching, planning and presenting adaptable social performance. Such early study has been supported by several subsequent studies (Minshew *et al.* 1992; Goldstein *et al.* 2001; Minshew & Goldstein, 2001; Ozonoff *et al.* 2004; Yerys *et al.* 2009). The most consistent findings are deficits in working memory (Barnard *et al.* 2008), planning (Ozonoff *et al.* 2004), and set-shifting (Minshew *et al.* 1992; Ozonoff *et al.* 2004) in ASD compared to typically developing (TD) individuals (Minshew *et al.* 1992; Ozonoff *et al.* 2004) or individuals with other neurodevelopmental disorders (Barnard *et al.* 2008).

With regards to working memory, individuals with ASD may have impaired spatial working memory (Williams *et al.* 2005; Russo *et al.* 2007; Steele *et al.* 2007), yet intact verbal working memory (Williams *et al.* 2005; Cui *et al.* 2010). The impaired spatial working memory in ASD is consistently evident across child (Goldberg *et al.* 2005; Williams *et al.* 2005; Corbett *et al.* 2009; Cui *et al.* 2010), adolescent (Steele *et al.* 2007) and adult (Williams *et al.* 2005) populations. Among the neuropsychological tests, the Spatial Span (SSP) and the Spatial Working Memory (SWM) of the Cambridge Neuropsychological Test Automated Battery (CANTAB; Goldberg *et al.* 2005; Corbett *et al.* 2009) have been used to assess working memory in children with ASD. Visuospatial planning is also impaired in children and adolescents (Hughes *et al.* 1994; Hughes, 1996; Ozonoff & Jensen, 1999; Geurts *et al.* 2004; Ozonoff *et al.* 2004, 2006; Landa & Goldberg, 2005) as well as adults (Barnard *et al.* 2008) with ASD. For example, Ozonoff *et al.* (2004) found youths with ASD took more thinking time and moves to complete trials on the Stockings of Cambridge task (SOC) than TD youths. Set-shifting reflecting cognitive flexibility is among the most consistent neuropsychological deficits in ASD, which is commonly assessed by the Wisconsin Card Sorting Test (WCST; Ozonoff & Jensen, 1999; Tsuchiya *et al.* 2005; Barnard *et al.* 2008; Kaland *et al.* 2008; Sumiyoshi *et al.* 2011) and the Intradimensional/Extradimensional Shift Test (I/ED) of the CANTAB (Hughes *et al.* 1994; Ozonoff *et al.* 2004; Corbett *et al.* 2009; Yerys *et al.* 2009).

The chronological change of executive function (EF) is of particular interest. Most studies have been conducted in samples with limited age range, except for a few studies including both children and adults with ASD (Williams *et al.* 2005; Happé *et al.* 2006; Rosenthal *et al.* 2013; Pugliese *et al.* 2014; Van den Bergh *et al.* 2014). Rosenthal and colleagues (2013) recruited 185 participants with ASD and divided them into four age groups (5–7, 8–10, 11–13, 14–18 years). Their results showed that older children with ASD had greater problems of initiating, working memory and organization than younger participants with ASD. Another study stratified ASD participants into four age groups (6–8, 9–11, 12–14, 15–18 years) and found that the impairments of inhibition and cognitive flexibility were most significant in childhood, while the planning deficit was apparent in early adolescence (12–14 years age group) (Van den Bergh *et al.* 2014). The findings of the study stratifying ASD participants into six age groups (4–5, 6–7, 8–9, 10–11, 12–13, 14–23 years) showed the EFs examined by the Behavior Rating Inventory of Executive Function, Parent Form was age-related and affected the performance of adaptive skills in children and youths with ASD (Pugliese *et al.* 2014). Happé *et al*. (2006) reported that the older subjects with ASD performed as well as normal controls on the tasks of response selection/inhibition, flexibility, and planning/working memory, suggesting an age-dependent improvement of EFs in ASD. However, most studies assessing planning ability in youths aged 9–15 showed worse performance in ASD than normal controls (Hughes *et al.* 1994; Hughes, 1996; Ozonoff & Jensen, 1999; Geurts *et al.* 2004; Ozonoff *et al.* 2004, 2006; Landa & Goldberg, 2005); similar deficits were also noted in adult ASD population (Barnard *et al.* 2008). Likewise, impaired working memory is consistently found across different age groups from children (Ozonoff & Jensen, 1999; Goldberg *et al.* 2005; Williams *et al.* 2005; Corbett *et al.* 2009; Cui *et al.* 2010), adolescents (Bennetto *et al.* 1996; Williams *et al.* 2005; Steele *et al.* 2007) to adults (Williams *et al.* 2005; Barnard *et al.* 2008) with ASD. In contrast, although youths with ASD aged 7–16 showed impaired set-shifting ability on the WCST (Ozonoff & Jensen, 1999; Tsuchiya *et al.* 2005; Kaland *et al.* 2008; Sumiyoshi *et al.* 2011) and CANTAB (Hughes *et al.* 1994; Ozonoff *et al.* 2004; Corbett *et al.* 2009; Yerys *et al.* 2009) compared to normal controls and clinical controls, such group difference in set-shifting ability was not demonstrated in the studies of adults with ASD on the WCST (Barnard *et al.* 2008; Sumiyoshi *et al.* 2011). Some of previous studies have proved the developmental importance of the period of adolescence that the synaptic pruning of excitatory contact happened in the late brain maturation during adolescence (Selemon, 2013), and the reduction of cortical thickness in the dorsal prefrontal region and maturity of gray-matter volume of temporal structure was found to be age-related during childhood to adulthood (Sowell *et al.* 2001). Luna and colleagues also found that age-related changes in establishing large-range connections could support more functional neural processing for maturing EFs (Luna *et al*. 2010). Adolescence is a critical period for brain development in TD and ASD populations as well (Begley, 2000; Giedd, 2008; Selemon, 2013), particularly the prefrontal systems which play a significant role in the executive processes (Luna *et al*. 2010). Related studies have shown evidence of age-related neurodevelopment from childhood to early adolescent in ASD (O'Hearn *et al.* 2008). EFs in this transition stage warrant investigation (Selemon, 2013).

Despite extensive research of EFs in ASD, the sample sizes of most studies are relatively too small to establish a conclusion (Ozonoff *et al.* 2004; Barnard *et al.* 2008; Sumiyoshi *et al.* 2011). Second, most studies recruited participants from a particular age range (Ozonoff *et al.* 2004; Barnard *et al.* 2008; Kaland *et al.* 2008; Corbett *et al.* 2009), and the chronological changes of executive dysfunctions in individuals with ASD are still unclear. Third, the relevant research is dominated by Western society (e.g. Goldberg *et al.* 2005; Ozonoff *et al.* 2006). Hence, we investigated the chronological difference of a wide range of EFs with varied task difficulty in a large-scale sample of Taiwanese youths with ASD and TD youths. Our hypothesis is that the impairments of EFs in youths with ASD will be moderated by age and task difficulty, and that the group difference in school age may decrease in adolescence and the group difference may increase with increased task difficulty.

## Method

### Participants and procedure

The Research Ethics Committee of National Taiwan University Hospital approved the study prior to the recruitment of participants (201201006RIB; ClinicalTrials.gov number, NCT01582256). We explained the purpose and procedure of the study, and obtained the written informed consent from all participants and their parents.

The sample consisted of 111 youths with clinical diagnosis of ASD (age 12.5 ± 2.8 years, male 94.6%) according to DSM-IVdiagnostic criteria of autistic disorder or Asperger's disorder (APA, 1994) from the child psychiatric clinics of National Taiwan University Hospital, and 114 TD youths (age 12.3 ± 2.3 years, male 94.7%) from schools and colleges referred by teachers according to the gender and age distribution of the ASD group. Among the ASD participants, 28 (25.22%) had ever been treated with methylphenidate, which had been discontinued at least 24 h before performing the tasks. Parents of both groups received the Kiddie Epidemiologic Version of the Schedule for Affective Disorders and Schizophrenia (K-SADS-E) to screen for any neuropsychiatric disorders (Gau *et al.* 2005). The interview training and best-estimate procedure have been described in detail elsewhere (Gau & Shang, 2010*a*; Shang & Gau, 2011).

The ASD diagnosis was further confirmed by interviewing parents of ASD youths using the Chinese version of the Autism Diagnostic Interview – Revised (ADI-R; Gau *et al.* 2011; Chen *et al.* 2014). Exclusion criteria for all participants included lifetime neurological or severe medical illness, learning disorder, substance use disorder, schizophrenia, bipolar disorder, major depression and current anxiety disorders. In order to complete the CANTAB, participants who were aged <8 years or who had full-scale IQ scores <80 were excluded from the study.

### Measures

#### ADI-R

The ADI-R is a standardized semi-structured assessment of the three core symptoms of autism in individuals aged >18 months including qualitative abnormalities in reciprocal social interaction, communication, and restricted interests and repetitive and stereotyped behaviors. The Chinese Version of the ADI-R was officially approved by Western Psychological Services (WPS) in May 2007 (Gau *et al.* 2011, 2013; Lau *et al.* 2013) and extensively used for clinical research in Taiwan (Gau *et al.* 2010; Chien *et al.* 2013*a*, *b*; Liao *et al.* 2013; Lin *et al.* 2013; Lo *et al.* 2013; Chen *et al.* 2014). All the interviewers reached agreement over 90%, ranging from 98.25 ± 1.91 to 99.38 ± 1.06 against the rating of each item in the Chinese ADI-R by a qualified ADI-R cross-site trainer (Y. Y. Wu) and on-site trainer (S. S. Gau) before implementation of this study.

#### Digit Span

Participants were instructed to repeat orally presented digits in order, or backward recall digits in the Wechsler Intelligence Scale for Children – 3rd edition (WISC-III). The Forward Digit Span test assesses verbal sustained attention and working memory, while the Backward Digit Span test assesses verbal working memory.

#### CANTAB

The CANTAB is a set of computerized tests to examine non-verbal neuropsychological functions (Robbins *et al.* 1998). Four subtests (SSP, SWM, SOC, I/ED) of the CANTAB were used to assess EFs (Gau & Shang, 2010*a*, *b*).

The SSP evaluates spatial short-term memory. At the start of the test the screen shows nine white boxes in fixed locations. Afterwards, the boxes change colors one after the other. Participants were asked to recall the sequence of color-changing boxes, and respond by touching boxes in order. The task begins with 2-box problems, and ends with 9-box problems if the participants are able to complete the task. This study presents span length (the longest sequence participants remember successfully) and total errors (the number of times participants choose incorrect boxes).

The SWM test for spatial working memory consists of three types of errors: ‘within error’ occurs when participants search the same box more than once in the same trial; ‘between error’ represents a mistake of searching boxes which have already shown blue tokens; and ‘double error’ can be categorized as both a within error and a between error. Two indexes are presented: strategy utilization (the number of searching strategies used in 6- and 8-box problems) and total errors in 4-, 6-, and 8-box problems (subtracting the number of double errors from the combination of within and between errors).

The SOC is used to evaluate planning ability. In this task, the screen is divided into upper and lower half parts. Each part presents three balls (red, blue, green) held in three suspended stockings. Participants were asked to plan and execute sequences of moves to achieve the desired arrangement. The two conditions are the *test condition*, in which participants were instructed to move balls in the lower part to match the arrangement in the upper part; and the *control condition*, which is designed to provide baseline measures for initiation time. This study presents problems solved in minimum moves (the number of times participants solved problems in the minimum number of moves) and ‘(2) “mean moves”’ (the number of moves within the range between minimum and maximum number of moves).

The I/ED is designed to evaluate mental flexibility. In this task, there are stimuli composed of unfamiliar pink shapes and white lines. Participants are instructed to select one of the two stimuli freely at the beginning. After they make the decision, the screen gives them feedbacks to assist in forming particular rules. The rules will be changed when participants have selected correct stimuli for six consecutive trials. To complete the task, participants have to pass through nine stages. The task ends if participants fail to select correct stimuli for 50 consecutive trials. This study presents (1) pre-extradimensional shift errors: participants select the correct stimuli depending on previously ignored dimension of stimuli; and (2) the number of completed stage trials.

### Data analyses

We used SAS v. 9.2 software (SAS Institute Inc., USA) to perform data analyses. To acquire descriptive data, categorical variables were presented with frequency and percentage and analyzed by χ^2^ test, the continuous variables were presented with mean and standard deviation (s.d.) and analyzed by analysis of variance (ANOVA) and analysis of covariance with age and sex as covariates for multivariate analyses.

To investigate whether the extent of worse EF performance in ASD than TD  individuals would vary between the child (age 8–12 years) and adolescent (age 13–18 years) groups, we tested the interactions between the diagnostic group and age group, and also conducted stratifying analysis by the child and adolescent groups. We also calculated Cohen's *d* for the effect size, which is graded by three levels, small (0.2–0.5), medium (0.5–0.8), and large (>0.8). To further investigate the effect of task difficulty, we used the mixed procedure of SAS to address the repeated measure within the same participants on the performances of the SWM and SOC.

## Results

### Sample characteristics

While the ages between adolescents with ASD and TD individuals were compatible, the mean ages between children with ASD and TD children were significantly different. Children with ASD had younger mean age (9.96 ± 1.37 years) and lower full-scale IQ (108.58 ± 15.97) than TD children (mean age 10.65 ± 1.31 years, IQ 114.94 ± 8.92) (Table 1). In general, the age, education level, and employment status of the parents were compatible between ASD and TD for both child and adolescent groups except for mothers’ age and employment status (Table 1).

**Table 1.** Demographics and IQ of youth with autism spectrum disorder (ASD) and typically developing (TD) youth, stratified by ages 8–12 and 13–18 years

	Age 8–12 years				Age 13–18 years			
	ASD (*N* = 53)	TD (*N* = 63)			ASD (*N* = 58)	TD (*N* = 51)		
	Mean (s.d.) or *N* (%)	Mean (s.d.) or *N* (%)	*F* value or χ^2^	*p* value	Mean (s.d.) or *N* (%)	Mean (s.d.) or *N* (%)	*F* value or χ^2^	*p* value
Age at assessment (years)	9.96 (1.37)	10.65 (1.31)	7.62	0.007**	14.72 (1.53)	14.41 (1.42)	1.21	0.274
Age range	8–12	8–12			13–18	13–18		
Gender								
Male	48 (90.6)	58 (92.1)	0.08	0.775	57 (98.28%)	50 (98.04%)	0.0084	0.927
Female	5 (9.4%)	5 (7.9%)			1 (1.72%)	1 (1.96%)		
Full-scale IQ	108.58 (15.97)	114.94 (8.92)	7.27	0.008**	107.07 (12.83)	109.92 (9.54)	1.70	0.196
Performance IQ	109.27 (16.34)	112.51 (12.27)	1.44	0.233	107.32 (16.03)	107.53 (13.00)	0.01	0.942
Verbal IQ	107.63 (17.29)	114.71 (9.01)	7.84	0.006**	107.14 (12.86)	110.80 (8.32)	2.99	0.087
Mother								
Current age	40.30 (4.23)	42.08 (4.38)	4.89	0.029*	44.81 (4.64)	44.82 (3.45)	0.00	0.987
Education level								
Senior high or lower	52 (98.11%)	63 (100.00%)	1.20	0.274	58 (100.00%)	51 (100.00%)	−	−
College or higher	1 (1.89%)	0 (0.00%)			0 (0.00%)	0 (0.00%)		
Employment status								
Professional	4 (7.55)	5 (7.94)	6.24	0.044*	5 (8.62%)	5 (10.00%)	0.17	0.920
Skilled work	23 (43.40)	41 (65.08)			30 (51.72%)	24 (48.00%)		
Others	26 (49.06)	17 (26.98)			23 (39.66%)	21 (42.00%)		
Father								
Current age	43.27 (5.19)	44.47 (4.80)	1.64	0.204	48.41 (4.99)	47.71 (4.20)	0.63	0.428
Education level								
Senior high or lower	51 (98.08%)	62 (100.00%)	1.20	0.273	58 (100.00%)	51 (100.00%)	−	−
College or higher	1 (1.92%)	0 (0.00%)			0 (0.00%)	0 (0.00%)		
Employment status								
Professional	9 (17.31%)	9 (14.52%)	1.54	0.462	14 (24.14%)	8 (16.00%)	2.25	0.324
Skilled work	41 (78.85%)	47 (75.81%)			35 (60.34%)	37 (74.00%)		
Others	2 (3.85%)	6 (9.68%)			9 (15.52%)	5 (10.00%)		
Autism Diagnostic Interview – Revised								
Current								
Qualitative abnormalities in reciprocal social interaction	10.53 (4.26)				8.50 (4.24)		6.30	0.014*
Qualitative abnormalities in communication (verbal)	10.12 (3.84)				13.67 (4.04)		2.34	0.135
Qualitative abnormalities in communication (non-verbal)	5.06 (2.41)				7.33 (1.53)		2.54	0.120
Restricted, repetitive, and stereotyped patterns of behavior	5.43 (2.44)				5.05 (2.66)		0.62	0.433
Most typical and severe at 4–5 years								
Qualitative abnormalities in reciprocal social interaction	20.11 (6.17)				18.02 (6.73)		2.91	0.091
Qualitative abnormalities in communication (verbal)	15.11 (3.37)				12.93 (4.83)		7.49	0.007**
Qualitative abnormalities in communication (non-verbal)	7.51 (2.19)				6.78 (3.35)		1.83	0.179
Restricted, repetitive, and stereotyped patterns of behavior	7.32 (2.45)				6.97 (2.85)		0.49	0.484
Abnormality of development evident at or before 36 month	3.17 (1.52)				2.60 (1.75)		3.30	0.072

IQ, Intelligence quotient; s.d., standard deviation.

* *p* < 0.05, **p < 0.01, ***p < 0.001.

### Age-stratified analysis (Table 2, Supplementary Table S1)

Both ASD groups recalled significantly fewer forward and backward digits than TD youths with large and medium effect sizes for the child (Cohen's *d* = −0.78, −0.98) and adolescent (Cohen's *d* = −0.68, −0.77) groups. Youths with ASD, regardless of age, recalled significantly shorter span sequences of SSP than TD youths (Cohen's *d* = −0.76, −0.60). For SWM, compared to TD youth, both ASD groups needed to use more strategies to complete their tasks (Cohen's *d* = 0.83, 0.78) but only adolescents with ASD had more total errors (Cohen's *d* = 0.51). For SOC, both ASD groups needed more total moves to complete the task (Cohen's *d* = 0.69, 0.42) than TD children but only children with ASD solved fewer problems in minimum moves (Cohen's *d* = −0.54). For I/ED, overall, the passing percentage of ASD youth fell strongly during the last two stages (Supplementary Fig. S1), yet only children with ASD made more pre-extra-dimensional shift errors (Cohen's *d* = 0.39) and completed fewer stages (Cohen's *d* = −0.37) than TD children without such group difference in the adolescent groups.

**Table 2.** Comparisons of executive functions among youth with autism spectrum disorder (ASD) and typically developing (TD) youth, stratified by ages 8–12 and 13–18 years

	Age 8–12 years				Age 13–18 years				Age effect	
Variables, mean (s.d.)	ASD (*N* = 53)	TD (*N* = 63)	Univariate analysis *F*(1,114)	Multivariate analysis *F*(3,112)^a^	ASD (*N* = 58)	TD (*N* = 51)	Univariate analysis *F*(1,107)	Multivariate analysis *F*(3,105)^a^	ASD *β* (*p* value)	TD *β* (*p* value)
Digit Span										
Digit Span, forward	7.63 (1.21)	8.46 (0.88)	17.72***	12.49***	8.11 (1.00)	8.67 (0.59)	11.80***	11.80***	0.10 (0.022)	0.08 (0.006)
Digit Span, backward	4.44 (1.57)	6.05 (1.70)	26.13***	20.171***	5.70 (1.71)	6.92 (1.44)	15.47***	15.81***	0.21 (0.001)	0.26 (<0.001)
Spatial Span										
Span length	6.04 (1.68)	7.14 (1.19)	17.15***	11.90***	7.16 (1.50)	7.98 (1.24)	9.67**	9.37**	0.19 (0.001)	0.25 (<0.001)
Total errors	14.02 (5.80)	14.65 (5.55)	0.35	0.96	12.48 (7.15)	11.16 (6.00)	1.08	1.36	−0.33 (0.138)	−0.90 (<0.001)
Spatial working memory										
Total errors	40.89 (19.85)	25.90 (15.99)	20.27***	13.07***	26.62 (17.98)	14.18 (13.62)	16.24***	16.91***	−2.85 (<0.001)	−3.53 (<0.001)
Strategy utilization	35.19 (4.25)	33.76 (3.66)	3.77	2.02	32.83 (5.61)	30.06 (5.33)	6.92**	7.70**	−0.48 (0.006)	−0.85 (<0.001)
Stockings of Cambridge										
Problems solved in minimum moves	6.92 (2.11)	8.02 (1.96)	8.30**	5.20*	8.81 (2.02)	9.37 (1.81)	2.31	2.46	0.31 (<0.001)	0.08 (<0.001)
Total moves	19.09 (2.73)	17.46 (1.94)	14.06***	11.11**	17.22 (2.26)	16.32 (1.97)	4.86*	4.94*	−0.30 (0.001)	−0.27 (0.001)
Intradimensional/extradimensional shift										
Extra-dimensional shift errors	12.74 (10.47)	10.16 (9.69)	1.89	1.34	7.97 (8.92)	7.53 (8.61)	0.07	0.08	−0.77 (0.023)	−0.65 (0.085)
Pre-extra-dimensional shift errors	8.40 (5.58)	6.75 (2.05)	4.76*	3.96*	6.81 (2.79)	7.31 (5.76)	0.35	0.41	−0.30 (0.042)	0.19 (0.255)
Completed stages	8.15 (1.17)	8.52 (0.80)	4.13*	3.97*	8.72 (0.67)	8.65 (1.13)	0.19	0.15	0.09 (0.007)	0.03 (0.473)

s.d., Standard deviation.

a Controlling for age and sex.

*p < 0.05, **p < 0.01, ***p < 0.001.

### Diagnosis, age and their interactions

For the whole sample, youths with ASD showed worse performance in the Digit Span, SSP, SWM, SOC, but not I/ED (Supplementary Table S1 and Table 2). There were significantly better performance in all the EF measures in the adolescent group than the child group.

### The effect of task difficulty

When testing the effect of task difficulty in the whole sample (Supplementary Table S3), we found significant interactions between the diagnosis group (ASD *v*. TD) and task difficulty on the number of total errors (6- *v*. 4-box problem, 8- *v*. 4-box problem) in SWM and on the mean moves (5- *v*. 2-move problem) in SOC.

**Fig. 1.**
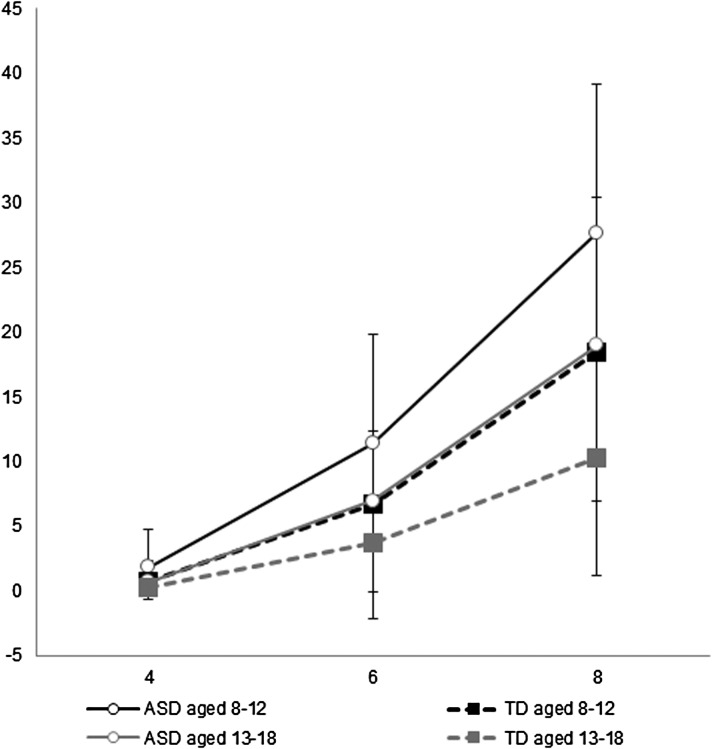
Total errors of the spatial working memory task for the ASD child and adolescent groups as well as the TD child and adolescent groups.

When stratified by age (Table 3), we found that the younger stratum showed significant interactions between the diagnosis group and all the difficulty levels of the SWM total errors; whereas, the elder stratum only revealed a significant interaction between diagnosis and the 8- *v*. 4-box problem. Fig. 1 also presents the magnitude of the difference between ASD and TD groups on the SWM total errors, enlarged as the task difficulty increased for the child and adolescent groups.

**Table 3.** A model integrating task difficulties, the diagnosis groups and their interactions, controlling for sex and age and stratifying by ages 8–12 and 13–18 years

	Age 8–12 years				Age 13–18 years			
	*β*	95% CI	*F* value	*p* value	*β*	95% CI	*F* value	*p* value
Spatial working memory (total errors)		*F*(1,228)				*F*(1,214)		
ASD *v*. control	−0.17	(−2.99 to 2.65)	0.01	0.904	0.60	(−2.18 to 3.37)	0.18	0.673
6- *v*. 4-boxes problem	5.95	(3.65 to 8.26)	79.86	<0.001***	3.43	(1.06–5.80)	34.58	<0.001***
8- *v*. 4-boxes problem	17.76	(15.45 to 20.07)	632.72	<0.001***	10.10	(7.73 to 12.47)	296.88	<0.001***
Group* (6- *v*. 4-box problem)	3.58	(0.17 to 6.99)	4.26	0.040*	2.83	(−0.42 to 6.08)	2.94	0.088
Group* (8- *v*. 4-box problem)	8.05	(4.64 to 11.46)	21.59	<0.001***	8.20	(4.95 to 11.44)	24.74	<0.001***
Stockings of Cambridge (mean moves)		*F*(1,342)				*F*(1,320)		
ASD *v*. control	0.09	(−0.27 to 0.45)	0.25	0.617	0.02	(−0.33 to 0.37)	0.01	0.906
3- *v*. 2-move problem	1.24	(0.93 to 1.55)	132.71	<0.001***	1.14	(0.80 to 1.48)	98.81	<0.001***
4- *v*. 2-move problem	3.35	(3.03 to 3.66)	907.97	<0.001***	3.18	(2.84 to 3.51)	717.92	<0.001***
5- *v*. 2-move problem	4.82	(4.51 to 5.13)	1906.59	<0.001***	4.00	(3.67 to 4.34)	1394.55	<0.001***
Group* (3- *v*. 2-move problem)	0.21	(−0.24 to 0.67)	0.85	0.359	0.07	(−0.39 to 0.53)	0.09	0.768
Group* (4- *v*. 2-move problem)	0.35	(−0.11 to 0.81)	2.22	0.137	−0.03	(−0.50 to 0.43)	0.02	0.885
Group* (5- *v*. 2-move problem)	0.56	(0.11 to 1.02)	5.84	0.016*	0.80	(0.33 to 1.26)	11.42	0.001

CI, confidence interval; *β*, regression coefficient estimates; ASD, autism spectrum disorder.

*p < 0.05, **p < 0.01, ***p < 0.001.

**Fig. 2.**
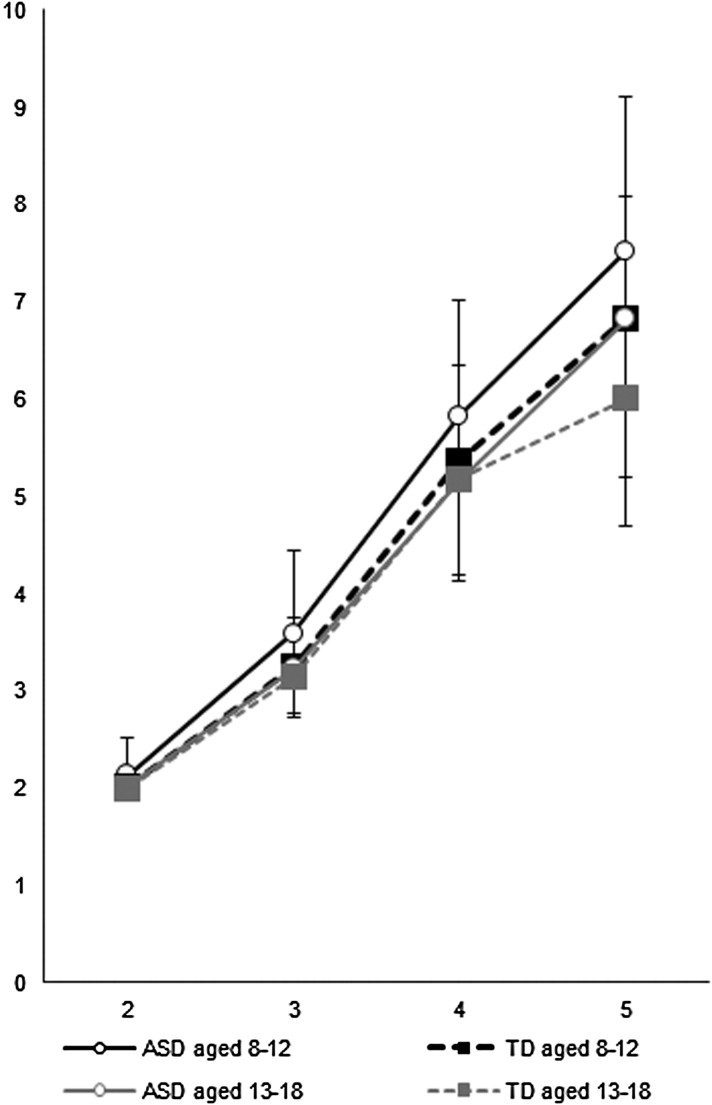
Total moves of the Stockings of Cambridge for the ASD child and adolescent groups as well as the TD child and adolescent groups.

In the mean moves of the SOC, there was significant interaction between the diagnosis and 5- *v*. 2-move problem for the child group (Table 3). Fig. 2 shows that the magnitude of the difference between ASD and TD groups on total moves of the SOC, enlarged as the task difficulty increased.

### The correlation between strategy utilization and the errors (Supplementary Table S5)

We further computed Pearson's correlation between strategy utilization and the number of errors of the SWM and found significant correlations between strategy utilization and three types of errors (total error, within error and between error) in both ASD and TD groups (Supplementary Table S5). Moreover, the correlations between errors and strategy use were greater with increasing level of task difficulty. The correlations did not significantly differ between ASD and TD groups (Supplementary Table S5)

### The correlation between EFs and ASD symptom severity in youth with ASD

To investigate the correlation between EFs and ASD symptom severity, we calculated Pearson's correlations to quantify the relationship between the CANTAB performance and the ADI-R subscores in youth with ASD for the child (Supplementary Table S6) and adolescent (Supplementary Table S7) groups. The results showed that while children with ASD showed significant correlations between the SOC and verbal communication deficits assessed by the ADI-R at the current and most severe status (Supplementary Table S6), there were significant correlations between the performance of the SOC and the verbal and non-verbal communication deficits assessed by the ADI-R at the most severe status in adolescents with ASD (Supplementary Table S7).

## Discussion

This study is the first to employ the comprehensive, standardized and computerized test battery, CANTAB, to assess EFs in an ethnic Chinese population, and is also one of the few studies recruiting a larger sample of ASD youths. The results demonstrated impaired EF in youths with ASD on short-term memory, spatial working memory, planning, and set-shifting, with an age-moderating effect mainly on planning and set-shifting, which were found impaired only in children with ASD but not in adolescents with ASD in our age-stratified analysis. We also found greater group difference with increased task difficulty in the SWM (Fig. 1) and SOC (Fig. 2) for both the child and adolescent groups.

Our findings of short-term spatial memory deficits in youth with ASD were consistent with some previous studies (Bowler *et al.* 1997; Minshew & Goldstein, 2001; Poirier *et al.* 2011) but not others (Prior & Chen, 1976; Williams *et al.* 2008). Such discrepant findings may be due to the different levels of task difficulty used across studies.

Compatible with the most previous studies showing impaired spatial working memory on various tasks in ASD (Williams *et al.* 2005, 2006; Joseph *et al.* 2005*a*; Gras-Vincendon *et al.* 2008; Kenworthy *et al.* 2008), our study demonstrated spatial working memory deficit in both age groups of ASD measured by the CANTAB. Some researchers explained the impairment by the utilization of strategy (Joseph *et al.* 2005*b*; Gras-Vincendon *et al.* 2008) that TD youth, but not youth with ASD, use verbal mediation strategies to locate positions in spatial working memory tasks. Nevertheless, there are few studies which recruit participants with high-functioning autism (Williams *et al.* 2005) or Asperger's disorder (Cui *et al.* 2010) showing no impairment of spatial working memory (Nakahachi *et al.* 1994; Williams *et al.* 2005, 2006). Functional level of participants with ASD is one possible reason for the inconsistency. Moreover, our finding of the impairment in both age groups is similar to the previous reports that individuals with ASD recalled shorter length or had more inefficient strategy utilization across the child and adolescent populations (Goldberg *et al.* 2005; Steele *et al.* 2007; Barnard *et al.* 2008; Corbett *et al.* 2009), suggesting that the spatial working memory deficit may persist into late adolescence as a trait marker for ASD.

Our findings of planning deficits in youths with ASD were similar to the results of most previous studies using either the same measurement (Ozonoff *et al.* 2004; Landa & Goldberg, 2005) or not (Ozonoff *et al.* 1991; Hughes *et al.* 1994; Ozonoff & Jensen, 1999; Geurts *et al.* 2004). Interestingly, some other studies evaluating planning by traditional measurements, such as the Tower of Hanoi (ToH) and the Tower of London (ToL), demonstrated that individuals with ASD had fewer passes and less efficient strategy than both healthy and comparison individuals with attention deficit hyperactivity disorder (Ozonoff *et al.* 1991; Hughes *et al.* 1994; Ozonoff & Jensen, 1999; Geurts *et al.* 2004). Regarding the age effect, although deficits in planning ability (total moves of SOC) were noted in both ASD groups with greater effect size in younger youths, only younger ASD youths demonstrated poorer problem-solving ability assessed by the SOC than their counterparts. Such findings indicate that ASD youths may improve their planning/problem-solving ability over time throughout adolescence. Our findings are in line with some evidence from previous studies of younger ASD individuals (Ozonoff *et al.* 1991; Hughes *et al.* 1994; Hughes, 1996; Ozonoff & Jensen, 1999; Geurts *et al.* 2004; Landa & Goldberg, 2005; Happé *et al.* 2006); whereas there is scarce evidence in older ASD individuals (Ozonoff *et al.* 2004). Hill's review concluded that planning ability in ASD was positively correlated with non-verbal mental age (El-Bassel *et al.* 2004) that the planning deficits were remarkable in childhood but would become lessen with age. In general, there was no significant diagnosis  ×  age interaction on SOC performance (Supplementary Table S2), suggesting a declined planning ability was noted in children, adolescents and young adults with ASD.

In contrast to previous studies which reported significant impairments of set-shifting in ASD as assessed by the I/ED of the CANTAB (Hughes *et al.* 1994; Ozonoff *et al.* 2004; Corbett *et al.* 2009; Yerys *et al.* 2009), the dimension-change card sort task (Dichter *et al.* 2010), WCST (Ozonoff & Jensen, 1999; Tsuchiya *et al.* 2005; Kaland *et al.* 2008; Sumiyoshi *et al.* 2011) and abstracting tests (Minshew *et al.* 1992), our findings only revealed poorer cognitive flexibility as assessed by the I/ED task in children with ASD (aged 8–12) but no difference in adolescents with ASD (aged 13–18). The observed discrepancies may be explained by different chronological age of the participants between studies. ASD participants recruited in previous studies were younger and showed more errors and different completion rates while performing the I/ED task (Hughes *et al.* 1994; Yerys *et al.* 2009; Dichter *et al.* 2010). However, findings in adolescents and young adults with ASD were inconsistent. Sumiyoshi *et al.* (2011) found that adults with ASD (mean age 26 years) performed significantly worse than TD individuals on the WCST. Another study did not find a significant difference (mean age 30 years) in comparison to participants with learning disabilities (Barnard *et al.* 2008). The discrepancy in adults with ASD may also be attributed to the nature of the set-shifting paradigms. Researchers have suggested that the widely administered measurement, i.e. the WCST, is more complex in that subjects have to recognize the patterns of the cards (including color, number, and shape), form abstract concepts, sustain the principle in mind and transfer the principle timely (Kaland *et al.* 2008), thus it evaluates functions more than set-shifting. In contrast, the CANTAB simplified the principles (i.e. line and figure) and presented them in a graded format, thus the results could acquire more reliable evidences of set-shifting ability (Robbins *et al.* 1998). Taken together, adults with ASD may not have set-shifting problems but that depends on which task was chosen. Whether adolescents with ASD have set-shifting problems warrants further investigation.

To summarize the chronological effect on EF, the results of current study showed impaired spatial working memory, planning, and set-shifting in children with ASD but improved in adolescents with ASD except for spatial working memory. Although the age-moderating effect is generally insignificant on planning and set-shifting, we found a trend that set-shifting ability in youth with ASD turned to parallel that of TD individuals as they grew up. Our findings were consistent with a few related studies that showed impaired cognitive flexibility and planning ability were age-dependent and apparent in childhood and early adolescence of ASD (Pugliese *et al.* 2014; Van den Bergh *et al.* 2014). Happé *et al.* (2006) also provided evidence that the performance of flexibility and planning in adolescents (aged 11–16) with ASD caught up with the performance of age-matched healthy controls. In line with one similar study investigating response preparation, inhibition and working memory in ASD samples (Luna *et al.* 2007), our findings imply that there were both typical and atypical developmental progressions of distinctive EFs in individuals with autism. The impaired EF such as planning and set-shifting which only presents in early childhood would imply a developmental delay and brain maturational processes which compensates for the inherent impairment (Luna *et al.* 2007). The study providing evidence of a reduction in cortical thickness in the dorsal prefrontal region between childhood and adolescence might explain a part of brain maturational processes and the related intact performance of planning and set-shifting task in older youth with ASD (Sowell *et al.* 2001). Instead of age-dependent planning and set-shifting ability, the persistent spatial working memory may serve as a trait marker for ASD and may nest in the core pathology of the ASD brain.

Consistent with previous studies using verbal working memory tasks (Rapin, 1996; Williams *et al.* 2006) or nonsense words (Gabig, 2008), we found a significant effect of task difficulty in both age stratifications that youths with ASD showed more errors on SWM relative to TD individuals when the tasks imposed greater demands, suggesting that spatial working memory deficits are fundamental in ASD regardless of task difficulty and aging effect (Fig. 1). Poor usage of compensatory strategies may lead to poorer performance. Although performing better in simple tasks, individuals with ASD failed to organize strategies to support memory in the tasks with increasing complexity of materials (Gras-Vincendon *et al.* 2008). Similarly, increased moves to complete the task were noted in SOC as the task demands increased, but planning deficits in ASD, relative to TD individuals, appeared only in the most difficult task (5-move problems) (Supplementary Table S3, Fig. 2). Unlike TD individuals who used fewer moves to solve problems, youths with ASD required more moves to complete the task, suggesting that the latter group tends to use trial-and-error, rather than taking time to develop an effective strategy before action. Other studies using the same tasks (Ozonoff *et al.* 2004) or the ToL task (Hughes *et al.* 1994; Geurts *et al.* 2004) also found that planning deficits were evident on trials requiring longer sequences of moves (4- and 5-move problems) in youths with ASD. Consistently, more discrepancy with increased task demands were also demonstrated in other planning paradigms such as a transferring task (Hughes, 1996), which requires subjects to pick up and transfer pegs from one hole to the other, and the Zoo Map test (Hill & Bird, 2006), which requires subjects to plan a route to destinations. More interestingly, when we stratified the sample by age, children with ASD used more moves across the four levels of task difficulty, while adolescents with ASD demonstrated planning deficits only in the most difficult task, implying that the task difficulty effect was more pronounced in older ASD youths rather than younger. Taken together, in contrast to the task difficulty effect in SWM regardless of age stratification, an interaction between age group and task difficulty was found in SOC, again implying that planning ability on simple tasks in ASD youths comes closer to that in the TD group, but impaired working memory persisted across developmental stages from childhood to adulthood. Hill's review article supports our statement that the planning ability in ASD participants improved as they became older, and the planning deficit was evident only on tasks requiring heavier demands (Hill, 2004).

Several limitations should be taken into consideration while interpreting our findings. First, due to a cross-sectional study design, we could only examine the age difference on EFs rather than the developmental trajectory of EFs. A longitudinal study is warranted to advance our knowledge about the chronological change of EFs. Second, the treatments effects on executive dysfunction were not considered in this study. Since most autistic participants in our study had received early intervention, the frequency and quantity of interventions would possibly affect participants’ performance, which should be inquired and controlled during data analyses. Last, this study only recruited subjects with an IQ >80 so they were able to perform the CANTAB tasks, our findings may not be applicable to ASD individuals with a lower IQ.

The findings in our study contribute not only to academia, but also to clinical practice. In past decades, with increasing government support and health education, the public has paid much closer attention to neurodevelopmental disorders. The interventions of ASD, in general, focus on improving communication and social skills, which are apparent and strongly educated in the public, while other functional domains that deserve more concerns have lacked attention. The results of our study provide evidence for caregivers and clinicians that some EFs (e.g. visuospatial planning and set-shifting) may improve to some extent in adolescence, yet working memory remains a weakness and warrants more coaching and assistance. Therapists may provide training aimed at strengthening specific EFs. Furthermore, considering the difficulties that increase with task demands, applying therapeutic activities with applicably graded task demands to both children and adolescents with ASD may potentially help increase the effect of interventions. For instance, providing activities such as asking children with ASD to recall words, sentences, and paragraphs in order could effectively improve their working memory ability (Rapin, 1996; Williams *et al.* 2006).

## Supplementary material

For supplementary material accompanying this paper visit http://dx.doi.org/10.1017/S0033291715002238. click here to view supplementary material

## References

[ref1] APA (1994). Diagnostic and Statistical Manual of Mental Disorder, 4th edn. American Psychiatric Press: Washington, DC.

[ref2] APA (2013). Diagnostic and Statistical Manual of Mental Disorders, 5th edn. American Psychiatric Association: Arlington, VA.

[ref3] Barnard L, Muldoon K, Hasan R, O'Brien G, Stewart M (2008). Profiling executive dysfunction in adults with autism and comorbid learning disability. Autism 12, 125–141. 18308763 10.1177/1362361307088486

[ref4] Baron-Cohen S (2002). The extreme male brain theory of autism. Trends in Cognitive Sciences 6, 248–254. 12039606 10.1016/s1364-6613(02)01904-6

[ref5] Baron-Cohen SE, Tager-Flusberg HE, Cohen DJ (2000). Understanding other Minds: Perspectives from Developmental Cognitive Neuroscience. Oxford University Press: Oxford.

[ref6] Baxter AJ, Brugha TS, Erskine HE, Scheurer RW, Vos T, Scott JG (2015). The epidemiology and global burden of autism spectrum disorders. Psychological Medicine 45, 601–613. 25108395 10.1017/S003329171400172X

[ref7] Begley S (2000). Getting inside a teen brain. Hormones aren't the only reason adolescents act crazy. Their gray matter differs from children's and adults’. Newsweek 135, 58–59. 10787984

[ref8] Bennetto L, Pennington BF, Rogers SJ (1996). Intact and impaired memory functions in autism. Child Development 67, 1816–1835. 8890510

[ref9] Blumberg SJ, Bramlett MD, Kogan MD, Schieve LA, Jones JR, Lu MC (2013). Changes in prevalence of parent-reported autism spectrum disorder in school-aged US children: 2007 to 2011–2012. National Health Statistics Reports 65, 1–11. 24988818

[ref10] Bowler DM, Matthews NJ, Gardiner JM (1997). Asperger's syndrome and memory: similarity to autism but not amnesia. Neuropsychologia 35, 65–70. 8981378 10.1016/s0028-3932(96)00054-1

[ref11] Chen CH, Huang CC, Cheng MC, Chiu YN, Tsai WC, Wu YY, Liu SK, Gau SS (2014). Genetic analysis of GABRB3 as a candidate gene of autism spectrum disorders. Molecular Autism 5, 36. 24999380 10.1186/2040-2392-5-36 PMC4082499

[ref12] Chien WH, Gau SS, Chen CH, Tsai WC, Wu YY, Chen PH, Shang CY, Chen CH (2013 *a*). Increased gene expression of FOXP1 in patients with autism spectrum disorders. Molecular Autism 4, 23. 23815876 10.1186/2040-2392-4-23 PMC3723673

[ref13] Chien WH, Gau SS, Liao HM, Chiu YN, Wu YY, Huang YS, Tsai WC, Tsai HM, Chen CH (2013 *b*). Deep exon resequencing of DLGAP2 as a candidate gene of autism spectrum disorders. Molecular Autism 4, 26. 23915500 10.1186/2040-2392-4-26 PMC3751063

[ref14] Corbett BA, Constantine LJ, Hendren R, Rocke D, Ozonoff S (2009). Examining executive functioning in children with autism spectrum disorder, attention deficit hyperactivity disorder and typical development. Psychiatry Research 166, 210–222. 19285351 10.1016/j.psychres.2008.02.005 PMC2683039

[ref15] Cui J, Gao D, Chen Y, Zou X, Wang Y (2010). Working memory in early-school-age children with Asperger's syndrome. Journal of Autism and Developmental Disorders 40, 958–967. 20108031 10.1007/s10803-010-0943-9

[ref16] Damasio A, Maurer R (1978). A neurological model for childhood autism. Archive of Neurology 35, 777–786. 10.1001/archneur.1978.00500360001001 718482

[ref17] Dawson G, Meltzoff AN, Osterling J, Rinaldi J (1998). Neuropsychological correlates of early symptoms of autism. Child Development 69, 1276–1285. 9839415 PMC4084601

[ref18] Dichter GS, Radonovich KJ, Turner-Brown LM, Lam KS, Holtzclaw TN, Bodfish JW (2010). Performance of children with autism spectrum disorders on the dimension-change card sort task. Journal of Autism and Developmental Disorders 40, 448–456. 19890707 10.1007/s10803-009-0886-1 PMC3709858

[ref19] El-Bassel N, Gilbert L, Frye V, Wu E, Go H, Hill J, Richman BL (2004). Physical and sexual intimate partner violence among women in methadone maintenance treatment. Psychology of Addictive Behaviors 18, 180–183. 15238060 10.1037/0893-164X.18.2.180

[ref20] Gabig CS (2008). Verbal working memory and story retelling in school-age children with autism. Language, Speech, and Hearing Services in Schools 39, 498. 10.1044/0161-1461(2008/07-0023) 18820091

[ref21] Gau SS, Chong MY, Chen TH, Cheng AT (2005). A 3-year panel study of mental disorders among adolescents in Taiwan. American Journal of Psychiatry 162, 1344–1350. 15994718 10.1176/appi.ajp.162.7.1344

[ref22] Gau SS, Liu LT, Wu YY, Chiu YN, Tsai WC (2013). Psychometric properties of the Chinese version of the social responsiveness scale. Research in Autism Spectrum Disorders 7, 349–360.

[ref23] Gau SS, Shang CY (2010 *a*). Executive functions as endophenotypes in ADHD: evidence from the Cambridge Neuropsychological Test Battery (CANTAB). Journal of Child Psychology and Psychiatry 51, 838–849. 20085608 10.1111/j.1469-7610.2010.02215.x

[ref24] Gau SS, Shang CY (2010 *b*). Improvement of executive functions in boys with attention deficit hyperactivity disorder: an open-label follow-up study with once-daily atomoxetine. International Journal of Neuropsychopharmacology 13, 243–256. 19849892 10.1017/S1461145709990836

[ref25] Gau SSF, Chou MC, Lee JC, Wong CC, Chou WJ, Chen MF, Soong WT, Wu YY (2010). Behavioral problems and parenting style among Taiwanese children with autism and their siblings. Psychiatry and Clinical Neurosciences 64, 70–78. 19968831 10.1111/j.1440-1819.2009.02034.x

[ref26] Gau SS-F, Lee C-M, Lai M-C, Chiu Y-N, Huang Y-F, Kao J-D, Wu Y-Y (2011). Psychometric properties of the Chinese version of the social communication questionnaire. Research in Autism Spectrum Disorders 5, 809–818.

[ref27] Geurts HM, Verté S, Oosterlaan J, Roeyers H, Sergeant JA (2004). How specific are executive functioning deficits in attention deficit hyperactivity disorder and autism? Journal of Child Psychology and Psychiatry 45, 836–854. 15056314 10.1111/j.1469-7610.2004.00276.x

[ref28] Giedd JN (2008). The teen brain: insights from neuroimaging. Journal of Adolescent Health 42, 335–343. 18346658 10.1016/j.jadohealth.2008.01.007

[ref29] Goldberg M, Mostofsky S, Cutting L, Mahone E, Astor B, Denckla M, Landa R (2005). Subtle executive impairment in children with autism and children with ADHD. Journal of Autism and Developmental Disorders 35, 279–293. 16119469 10.1007/s10803-005-3291-4

[ref30] Goldstein G, Johnson CR, Minshew NJ (2001). Attentional processes in autism. Journal of Autism and Developmental Disorders 31, 433–440. 11569589 10.1023/a:1010620820786

[ref31] Gras-Vincendon A, Bursztejn C, Danion JM (2008). Functioning of memory in subjects with autism. Encephale 34, 550–556. 19081450 10.1016/j.encep.2007.10.010

[ref32] Happé F, Booth R, Charlton R, Hughes C (2006). Executive function deficits in autism spectrum disorders and attention-deficit/hyperactivity disorder: examining profiles across domains and ages. Brain and Cognition 61, 25–39. 16682102 10.1016/j.bandc.2006.03.004

[ref33] Hill EL (2004). Evaluating the theory of executive dysfunction in autism. Developmental Review 24, 189–233.

[ref34] Hill EL, Bird CM (2006). Executive processes in Asperger syndrome: patterns of performance in a multiple case series. Neuropsychologia 44, 2822–2835. 16930637 10.1016/j.neuropsychologia.2006.06.007

[ref35] Hughes C (1996). Brief report: planning problems in autism at the level of motor control. Journal of Autism and Developmental Disorders 26, 99–107. 8819773 10.1007/BF02276237

[ref36] Hughes C, Russell J, Robbins TW (1994). Evidence for executive dysfunction in autism. Neuropsychologia 32, 477–492. 8047253 10.1016/0028-3932(94)90092-2

[ref38] Joseph RM, McGrath LM, Tager-Flusberg H (2005 *a*). Executive dysfunction and its relation to language ability in verbal school-age children with autism. Developmental Neuropsychology 27, 361–378. 15843102 10.1207/s15326942dn2703_4 PMC1201456

[ref39] Joseph RM, Steele SD, Meyer E, Tager-Flusberg H (2005 *b*). Self-ordered pointing in children with autism: failure to use verbal mediation in the service of working memory? Neuropsychologia 43, 1400–1411. 15989932 10.1016/j.neuropsychologia.2005.01.010

[ref40] Kaland N, Smith L, Mortensen EL (2008). Brief report: cognitive flexibility and focused attention in children and adolescents with Asperger syndrome or high-functioning autism as measured on the computerized version of the Wisconsin Card Sorting Test. Journal of Autism and Developmental Disorders 38, 1161–1165. 17965928 10.1007/s10803-007-0474-1

[ref41] Kenworthy L, Yerys BE, Anthony LG, Wallace GL (2008). Understanding executive control in autism spectrum disorders in the lab and in the real world. Neuropsychology Review 18, 320–338. 18956239 10.1007/s11065-008-9077-7 PMC2856078

[ref42] Lai DC, Tseng YC, Guo HR (2013). Trends in the prevalence of childhood disability: analysis of data from the national disability registry of Taiwan, 2000–2011. Research in Developmental Disabilities 34, 3766–3772. 24021391 10.1016/j.ridd.2013.08.001

[ref43] Lai DC, Tseng YC, Hou YM, Guo HR (2012). Gender and geographic differences in the prevalence of autism spectrum disorders in children: analysis of data from the National Disability Registry of Taiwan. Research in Developmental Disabilities 33, 909–915. 22245733 10.1016/j.ridd.2011.12.015

[ref44] Landa RJ, Goldberg MC (2005). Language, social, and executive functions in high functioning autism: a continuum of performance. Journal of Autism and Developmental Disorders 35, 557–573. 16211332 10.1007/s10803-005-0001-1

[ref45] Lau WY, Gau SS, Chiu YN, Wu YY, Chou WJ, Liu SK, Chou MC (2013). Psychometric properties of the Chinese version of the autism spectrum quotient (AQ). Research in Developmental Disabilities 34, 294–305. 22985783 10.1016/j.ridd.2012.08.005

[ref46] Liao HM, Gau SS, Tsai WC, Fang JS, Su YC, Chou MC, Liu SK, Chou WJ, Wu YY, Chen CH (2013). Chromosomal abnormalities in patients with autism spectrum disorders from Taiwan. American Journal of Medical Genetics, B: Neuropsychiatric Genetics 162B, 734–741. 24132905 10.1002/ajmg.b.32153

[ref47] Lin LY (2015). Coping strategies, caregiving burden, and depressive symptoms of Taiwanese mothers of adolescents with autism spectrum disorder. Research in Autism Spectrum Disorders 15, 1–9.

[ref48] Lin PI, Kuo PH, Chen CH, Wu JY, Gau SS, Wu YY, Liu SK (2013). Runs of homozygosity associated with speech delay in autism in a Taiwanese Han population: evidence for the recessive model. PLoS ONE 8, . 10.1371/journal.pone.0072056 PMC3745408 23977206

[ref49] Lo YC, Chou TL, Fan LY, Gau SS, Chiu YN, Tseng WY (2013). Altered structure-function relations of semantic processing in youths with high-functioning autism: a combined diffusion and functional MRI Study. Autism Research 12, 561–570. 23853172 10.1002/aur.1315

[ref50] Luna B, Doll SK, Hegedus SJ, Minshew NJ, Sweeney JA (2007). Maturation of executive function in autism. Biological Psychiatry 61, 474–481. 16650833 10.1016/j.biopsych.2006.02.030

[ref51] Luna B, Padmanabhan A, O'Hearn K (2010). What has fMRI told us about the development of cognitive control through adolescence? Brain and Cognition 72, 101–113. 19765880 10.1016/j.bandc.2009.08.005 PMC2815087

[ref52] Martin A, Volkmar FR, Lewis M (eds) (2007). Lewis's Child and Adolescent Psychiatry: A Comprehensive Textbook. Lippincott Williams & Wilkins: Philadelphia.

[ref53] Matson JL, Kozlowski AM (2011). The increasing prevalence of autism spectrum disorders. Research in Autism Spectrum Disorders 5, 418–425.

[ref55] Minshew NJ, Goldstein G (2001). The pattern of intact and impaired memory functions in autism. Journal of Child Psychology and Psychiatry 42, 1095–1101. 11806691 10.1111/1469-7610.00808

[ref56] Minshew NJ, Muenz LR, Goldstein G, Payton JB (1992). Neuropsychological functioning in nonmentally retarded autistic individuals. Journal of Clinical and Experimental Neuropsychology 14, 749–761. 1474143 10.1080/01688639208402860

[ref57] Nakahachi T, Iwase M, Takahashi H, Honaga E, Sekiyama R, Ukai S, Ishii R, Osterling J, Dawson G (1994). Early recognition of children with autism: a study of first birthday home videotapes. Journal of Autism and Developmental Disorders 24, 247–257. 8050980 10.1007/BF02172225

[ref58] O'Hearn K, Asato M, Ordaz S, Luna B (2008). Neurodevelopment and executive function in autism. Development and Psychopathology 20, 1103–1132. 18838033 10.1017/S0954579408000527

[ref58a] Osterling J, Dawson G (1994). Early recognition of children with autism: a study of first birthday home videotapes. Journal of Autism and Developmental Disorders 24, 247–257. 8050980 10.1007/BF02172225

[ref59] Ozonoff S, Cook I, Coon H, Dawson G, Joseph RM, Klin A, McMahon WM, Minshew N, Munson JA, Pennington BF (2004). Performance on Cambridge Neuropsychological Test Automated Battery subtests sensitive to frontal lobe function in people with autistic disorder: evidence from the Collaborative Programs of Excellence in Autism network. Journal of Autism and Developmental Disorders 34, 139–150. 15162933 10.1023/b:jadd.0000022605.81989.cc

[ref60] Ozonoff S, Jensen J (1999). Brief report: specific executive function profiles in three neurodevelopmental disorders. Journal of Autism and Developmental Disorders 29, 171–177. 10382139 10.1023/a:1023052913110

[ref61] Ozonoff S, Pennington BF, Rogers SJ (2006). Executive function deficits in high-functioning autistic individuals: relationship to theory of mind. Journal of Child Psychology and Psychiatry 32, 1081–1105. 1787138 10.1111/j.1469-7610.1991.tb00351.x

[ref62] Ozonoff S, Rogers SJ, Pennington BF (1991). Asperger's syndrome: evidence of an empirical distinction from high-functioning Autism. Journal of Child Psychology and Psychiatry 32, 1107–1122. 1787139 10.1111/j.1469-7610.1991.tb00352.x

[ref63] Poirier M, Martin JS, Gaigg SB, Bowler DM (2011). Short-term memory in autism spectrum disorder. Journal of Abnormal Psychology 120, 247. 21319933 10.1037/a0022298

[ref64] Pugliese CE, Anthony L, Strang JF, Dudley K, Wallace GL, Kenworthy L (2014). Increasing adaptive behavior skill deficits from childhood to adolescence in autism spectrum disorder: role of executive function. Journal of Autism and Developmental Disorders 45, 1579–1587. 25398602 10.1007/s10803-014-2309-1 PMC4433442

[ref65] Prior MR, Chen C (1976). Short-term and serial memory in autistic, retarded, and normal children. Journal of Autism and Childhood Schizophrenia 6, 121–131. 134993 10.1007/BF01538055

[ref66] Rapin I (1996). Preschool Children with Inadequate Communication: Developmental Language Disorder, Autism, Low IQ. Mac Keith Press: Suffolk.

[ref67] Robbins TW, James M, Owen AM, Sahakian BJ, Lawrence AD, McInnes L, Rabbitt PM (1998). A study of performance on tests from the CANTAB battery sensitive to frontal lobe dysfunction in a large sample of normal volunteers: implications for theories of executive functioning and cognitive aging. Journal of the International Neuropsychological Society 4, 474–490. 9745237 10.1017/s1355617798455073

[ref68] Robinson S, Goddard L, Dritschel B, Wisley M, Howlin P (2009). Executive functions in children with autism spectrum disorders. Brain and Cognition 71, 362–368. 19628325 10.1016/j.bandc.2009.06.007

[ref69] Rosenthal M, Wallace GL, Lawson R, Wills MC, Dixon E, Yerys BE, Kenworthy L (2013). Impairments in real-world executive function increase from childhood to adolescence in autism spectrum disorders. Neuropsychology 27, 13. 23356593 10.1037/a0031299 PMC4747021

[ref70] Russo N, Flanagan T, Iarocci G, Berringer D, Zelazo PD, Burack JA (2007). Deconstructing executive deficits among persons with autism: implications for cognitive neuroscience. Brain and Cognition 65, 77–86. 17825970 10.1016/j.bandc.2006.04.007

[ref71] Salmond C, De Haan M, Friston K, Gadian D, Vargha-Khadem F (2003). Investigating individual differences in brain abnormalities in autism. Philosophical Transactions of the Royal Society of London. Series B: Biological Sciences 358, 405–413. 12639337 10.1098/rstb.2002.1210 PMC1693120

[ref72] Selemon LD (2013). A role for synaptic plasticity in the adolescent development of executive function. Translational Psychiatry 3, e238. 23462989 10.1038/tp.2013.7 PMC3625918

[ref73] Shang CY, Gau SS (2011). Visual memory as a potential cognitive endophenotype of attention deficit hyperactivity disorder. Psychological Medicine 40, 2603–2614. 21733210 10.1017/S0033291711000857

[ref74] Sowell ER, Delis D, Stiles J, Jernigan TL (2001). Improved memory functioning and frontal lobe maturation between childhood and adolescence: a structural MRI study. Journal of the International Neuropsychological Society 7, 312–322. 11311032 10.1017/s135561770173305x

[ref75] Steele SD, Minshew NJ, Luna B, Sweeney JA (2007). Spatial working memory deficits in autism. Journal of Autism and Developmental Disorders 37, 605–612. 16909311 10.1007/s10803-006-0202-2

[ref76] Sumiyoshi C, Kawakubo Y, Suga M, Sumiyoshi T, Kasai K (2011). Impaired ability to organize information in individuals with autism spectrum disorders and their siblings. Neuroscience Research 69, 252–257. 21129422 10.1016/j.neures.2010.11.007

[ref77] Tsuchiya E, Oki J, Yahara N, Fujieda K (2005). Computerized version of the Wisconsin card sorting test in children with high-functioning autistic disorder or attention-deficit/hyperactivity disorder. Brain and Development 27, 233–236. 15737707 10.1016/j.braindev.2004.06.008

[ref78] Van den Bergh SF, Scheeren AM, Begeer S, Koot HM, Geurts HM (2014). Age related differences of executive functioning problems in everyday life of children and adolescents in the autism spectrum. Journal of Autism and Developmental Disorders 44, 1959–1971. 24562693 10.1007/s10803-014-2071-4

[ref79] Williams D, Happé F, Jarrold C (2008). Intact inner speech use in autism spectrum disorder: evidence from a short-term memory task. Journal of Child Psychology and Psychiatry 49, 51–58. 18181880 10.1111/j.1469-7610.2007.01836.x

[ref80] Williams DL, Goldstein G, Carpenter PA, Minshew NJ (2005). Verbal and spatial working memory in autism. Journal of Autism and Developmental Disorders 35, 747–756. 16267641 10.1007/s10803-005-0021-x

[ref81] Williams DL, Goldstein G, Minshew NJ (2006). The profile of memory function in children with autism. Neuropsychology 20, 21. 16460219 10.1037/0894-4105.20.1.21 PMC1847594

[ref82] Yerys BE, Wallace GL, Harrison B, Celano MJ, Giedd JN, Kenworthy LE (2009). Set-shifting in children with autism spectrum disorders reversal shifting deficits on the Intradimensional/Extradimensional Shift Test correlate with repetitive behaviors. Autism 13, 523–538. 19759065 10.1177/1362361309335716 PMC3018342

