# Supplementary material for: Deficits in executive functions among youths with autism spectrum disorders: an age-stratified analysis
Source: Psychol Med. 2016 Mar 21;46(8):1625–38. doi: 10.1017/S0033291715002238 (PMC4873936; doi:10.1017/S0033291715002238)
Supplement: Supplementary file 1 [file S0033291715002238sup.zip › S0033291715002238sup002.docx]

**Supplementary Table S1.** *Comparisons of executive functions among youth with autism spectrum disorder and typically developing youth: present with Cohen’s d*

| Variables, Mean (SD) |  | | | | Ages 8-12 | | | Ages 13-18 | | |  |
| --- | --- | --- | --- | --- | --- | --- | --- | --- | --- | --- | --- |
|  | ASD  (N=111) | TD  (N=114) | | Cohen’s d | ASD  (N=53) | TD  (N=63) | Cohen’s d | ASD  (N=58) | TD  (N=51) | Cohen’s d |  |
| **Digit Span** |  |  | |  |  |  |  |  |  |  |  |
| Digit Span, forward | 7.88(1.13) | 8.55(0.77) | | -0.69 | 7.63 (1.21) | 8.46 (0.88) | -0.78 | 8.11(1.00) | 8.67 (0.59) | -0.68 |  |
| Digit Span, backward | 5.11(1.76) | 6.44(1.64) | | -0.78 | 4.44 (1.57) | 6.05 (1.70) | -0.98 | 5.70(1.71) | 6.92 (1.44) | -0.77 |  |
| **Spatial Span** |  |  | |  |  |  |  |  |  |  |  |
| Span Length | 6.62(1.67) | 7.52(1.28) | | -0.60 | 6.04 (1.68) | 7.14 (1.19) | -0.76 | 7.16(1.50) | 7.98 (1.24) | -0.60 |  |
| Total errors | 13.22(6.56) | 13.07(5.99) | | 0.02 | 14.02 (5.80) | 14.65 (5.55) | -0.11 | 12.48(7.15) | 11.16 (6.00) | 0.20 |  |
| **Spatial Working Memory** | |  | |  |  |  |  |  |  |  |  |
| Total errors | 33.43(20.12) | 20.66(16.02) | | 0.70 | 40.89 (19.85) | 25.90 (15.99) | 0.83 | 26.62(17.98) | 14.18 (13.62) | 0.78 |  |
| Strategy Utilization | 33.95(5.12) | 32.11(4.83) | | 0.37 | 35.19 (4.25) | 33.76 (3.66) | 0.36 | 32.83(5.61) | 30.06 (5.33) | 0.51 |  |
| **Stockings of Cambridge** |  |  | |  |  |  |  |  |  |  |  |
| Problems solved in minimum moves | 7.91(2.26) | 8.62(2.01) | | -0.33 | 6.92 (2.11) | 8.02 (1.96) | -0.54 | 8.81(2.02) | 9.37 (1.81) | -0.29 |  |
| Total moves | 18.11(2.65) | 16.95(2.03) | | 0.49 | 19.09 (2.73) | 17.46 (1.94) | 0.69 | 17.22(2.26) | 16.32 (1.97) | 0.42 |  |
| **Intradimensional/Extradimensional shift** | | | |  |  |  |  |  |  |  |  |
| Extra-dimensional shift errors | 10.24(9.94) | | 8.98(9.28) | 0.13 | 12.74(10.47) | 10.16(9.69) | 0.26 | 7.97(8.92) | 7.53(8.61) | 0.05 |  |
| Pre-extra-dimensional shift errors | 7.57(4.40) | 7.00(4.13) | | 0.13 | 8.40 (5.58) | 6.75 (2.05) | 0.39 | 6.81(2.79) | 7.31(5.76) | -0.11 |  |
| Completed stages | 8.45(0.98) | 8.58(0.96) | | -0.13 | 8.15 (1.17) | 8.52 (0.80) | -0.37 | 8.72(0.67) | 8.65(1.13) | 0.08 |  |

Note: SD = standard deviation; ASD = autism spectrum disorder; TD = typically developing youth;

🟅Controlling for age and sex; *p < .05; ** p < .01; *** p < .001

**Supplementary Table S2.** *The effects of group, age group and group x age group*

| Variables, Mean(SD) |  |  |  |  | Diagnostic group (ASD vs. TD)  (F value) | Age group  (Ages 13-18 vs. Ages 8-12)  (F value) | Diagnosis*Age  (F value) |
| --- | --- | --- | --- | --- | --- | --- | --- |
| **Digit Span (Verbal Executive Functioning)** | | |  |  |  |  |  |
| Digit Span, forward |  |  |  |  | 29.22*** | 7.24** | 1.18 |
| Digit Span, backward |  |  |  |  | 40.93*** | 23.44*** | 0.79 |
| **Spatial Span** |  |  |  |  |  |  |  |
| Span length |  |  |  |  | 26.25*** | 26.92*** | 0.55 |
| Total errors |  |  |  |  | 0.18 | 9.26** | 1.40 |
| **Spatial Working Memory** | |  |  |  |  |  |  |
| Total errors |  |  |  |  | 36.27*** | 32.58*** | 0.31 |
| Strategy Utilization |  |  |  |  | 10.89** | 22.76*** | 1.11 |
| **Stockings of Cambridge** | |  |  |  |  |  |  |
| Problems solved in minimum moves |  |  |  |  | 9.73** | 37.41*** | 1.00 |
| Total moves |  |  |  |  | 17.89*** | 25.47*** | 1.48 |
| **Intradimensional/Extradimensional shift** | | |  |  |  |  |  |
| Extra-dimensional shift errors |  |  |  |  | 1.42 | 8.55** | 0.72 |
| Pre-extra-dimensional shift errors |  |  |  |  | 1.02 | 0.80 | 3.60 |
| Completed stages |  |  |  |  | 1.35 | 7.50** | 3.13 |

Note: SD = standard deviation; group = youth with autism spectrum disorder versus typically developing youth; age group = age 8-12 versus age 13 and older

*p < .05; ** p < .01; *** p < .001.

**Supplementary Table S3.** *A model integrating task difficulties, diagnosis group and their interaction, controlling for age and sex*

|  | β | 95%CI | F value | P value🟅 |
| --- | --- | --- | --- | --- |
| **Spatial Working Memory** |  |  |  |  |
| Total errors |  |  | F(1,446) |  |
| ASD vs. TD | 0.86 | (-1.15, 2.86) | 0.71 | 0.401 |
| 6- vs. 4-boxes problem | 4.82 | (3.11, 6.54) | 103.48 | <.001*** |
| 8- vs. 4-boxes problem | 14.33 | (12.62, 16.05) | 848.90 | <.001*** |
| group *(6- vs. 4-boxes problem) | 3.00 | (0.55, 5.44) | 5.81 | 0.016* |
| group *(8- vs. 4-boxes problem) | 7.55 | (5.11, 9.99) | 36.89 | <.001*** |
| **Stockings of Cambridge** |  |  |  |  |
| **Mean moves** |  |  | F(1,669) |  |
| ASD vs.TD | 0.08 | (-0.17, 0.32) | 0.35 | 0.5529 |
| 3- vs. 2-move problem | 1.19 | (0.96, 1.42) | 225.55 | <.001*** |
| 4- vs. 2-move problem | 3.27 | (3.04, 3.50) | 1585.97 | <.001*** |
| 5- vs. 2-move problem | 4.45 | (4.22, 4.69) | 3234.54 | <.001*** |
| group*(3- vs. 2-move problem) | 0.13 | (-0.20, 0.46) | 0.61 | 0.4336 |
| group*(4- vs. 2-move problem) | 0.14 | (-0.19, 0.46) | 0.66 | 0.4186 |
| group*(5- vs. 2-move problem) | 0.62 | (0.30, 0.95) | 13.90 | <.001*** |

Note: CI = confidence interval; β = regression coefficient estimates

*p<0.05 **p<0.01 ***p<0.001

**Supplementary Table S4.** *Comparisons of executive functions among youth with autism spectrum disorder and typically developing youth: stratified by ages of 8-12 and ages of 13-18* ( *controlling for sex, age and IQ )*

| Variables, Mean (SD) | Ages 8-12 | | | |  | Ages 13-18 | | | |  |
| --- | --- | --- | --- | --- | --- | --- | --- | --- | --- | --- |
|  | ASD  (N=53) | TD  (N=63) | Univariate analysis  F (1,114) | Multivariate analysis🟅  F(3,112) | Multivariate analysis🟅🟅  F(4,111) | ASD  (N=58) | TD  (N=51) | Univariate analysis  F (1,107 ) | Multivariate analysis🟅  F (3,105) | Multivariate analysis🟅🟅  F (4,104) |
| **Digit Span** |  |  |  |  |  |  |  |  |  |  |
| Digit Span, forward | 7.63 (1.21) | 8.46 (0.88) | 17.72*** | 12.49*** | 8.39** | 8.11(1.00) | 8.67 (0.59) | 11.80*** | 11.80*** | 10.05** |
| Digit Span, backward | 4.44 (1.57) | 6.05 (1.70) | 26.13*** | 20.171*** | 15.89*** | 5.70(1.71) | 6.92 (1.44) | 15.47*** | 15.81*** | 14.18*** |
| **Spatial Span** |  |  |  |  |  |  |  |  |  |  |
| Span Length | 6.04 (1.68) | 7.14 (1.19) | 17.15*** | 11.90*** | 6.03* | 7.16(1.50) | 7.98 (1.24) | 9.67** | 9.37** | 8.01** |
| Total errors | 14.02 (5.80) | 14.65 (5.55) | 0.35 | 0.96 | 1.99 | 12.48(7.15) | 11.16 (6.00) | 1.08 | 1.36 | 0.98 |
| **Spatial Working Memory** | |  |  |  |  |  |  |  |  |  |
| Total errors | 40.89 (19.85) | 25.90 (15.99) | 20.27*** | 13.07*** | 6.77* | 26.62(17.98) | 14.18 (13.62) | 16.24*** | 16.91*** | 15.48*** |
| Strategy Utilization | 35.19 (4.25) | 33.76 (3.66) | 3.77 | 2.02 | 0.75 | 32.83(5.61) | 30.06 (5.33) | 6.92** | 7.70** | 6.76* |
| **Stockings of Cambridge** |  |  |  |  |  |  |  |  |  |  |
| Problems solved in minimum moves | 6.92 (2.11) | 8.02 (1.96) | 8.30** | 5.20* | 2.36 | 8.81(2.02) | 9.37 (1.81) | 2.31 | 2.46 | 1.76 |
| Total moves | 19.09 (2.73) | 17.46 (1.94) | 14.06*** | 11.11** | 5.88* | 17.22(2.26) | 16.32 (1.97) | 4.86* | 4.94* | 3.99* |
| **Intradimensional/Extradimensional shift** | | |  |  |  |  |  |  |  |  |
| Extra-dimensional shift errors | 12.74(10.47) | 10.16(9.69) | 1.89 | 1.34 | 1.93 | 7.97(8.92) | 7.53(8.61) | 0.07 | 0.08 | 0.01 |
| Pre-extra-dimensional shift errors | 8.40 (5.58) | 6.75 (2.05) | 4.76* | 3.96* | 1.68 | 6.81(2.79) | 7.31(5.76) | 0.35 | 0.41 | 0.39 |
| Completed stages | 8.15 (1.17) | 8.52 (0.80) | 4.13* | 3.97* | 1.81 | 8.72(0.67) | 8.65(1.13) | 0.19 | 0.15 | 0.13 |

🟅 Controlling for age and sex; 🟅🟅Controlling for age, sex and IQ; *p < .05; ** p < .01; *** p < .001

**Supplementary Table S5.**  *Correlations between strategy utilization and the number of errors of the SWM*

| Pearson Correlation Coefficients | ASD  (N=111) | | TD  (N=114) | | Fisher Z test | |
| --- | --- | --- | --- | --- | --- | --- |
|  | R | P value | R | P value | z-score | P value |
| Total errors | 0.70 | <.001*** | 0.64 | <.001*** | 0.81 | 0.210 |
| 4 box problems | 0.22 | 0.022* | 0.25 | 0.007** | -0.24 | 0.407 |
| 6 box problems | 0.61 | <.001*** | 0.53 | <.001*** | 0.88 | 0.190 |
| 8 box problems | 0.69 | <.001*** | 0.60 | <.001*** | 1.15 | 0.126 |
| Within errors | 0.32 | 0.001*** | 0.21 | 0.028* | 0.88 | 0.190 |
| 4 box problems | 0.18 | 0.063 | 0.16 | 0.088 | 0.15 | 0.439 |
| 6 box problems | 0.27 | 0.004** | 0.19 | 0.047* | 0.63 | 0.266 |
| 8 box problems | 0.22 | 0.021 | 0.13 | 0.184 | 0.69 | 0.246 |
| Between errors | 0.69 | <.001*** | 0.64 | <.001*** | 0.66 | 0.253 |
| 4 box problems | 0.21 | 0.026* | 0.24 | 0.009** | -0.23 | 0.408 |
| 6 box problems | 0.60 | <.001*** | 0.53 | <.001*** | 0.76 | 0.223 |
| 8 box problems | 0.69 | <.001*** | 0.60 | <.001*** | 1.15 | 0.126 |
| Double errors | 0.27 | 0.005** | 0.19 | 0.049* | 0.63 | 0.266 |
| 4 box problems | 0.11 | 0.238 | - | - | 0.82 | 0.207 |
| 6 box problems | 0.33 | 0.000*** | 0.22 | 0.018** | 0.88 | 0.189 |
| 8 box problems | 0.16 | 0.089 | 0.12 | 0.198 | 0.30 | 0.381 |

*p<.05 **p<.01 ***p<.001

**Supplementary Table S6.** *The correlation between the performance of CANTAB and ADI-R: Age 8-12 (N=53)*

| Pearson Correlation Coefficients (r) | **ADIR** | | | | | | | | |  |
| --- | --- | --- | --- | --- | --- | --- | --- | --- | --- | --- |
|  | Current | | | | Severe | | | | |  |
|  | Reciprocal social interaction | Communication  (verbal) | Communication  (nonverbal) | Restricted, repetitive, and stereotyped patterns of behavior | Reciprocal social interaction | Communication  (verbal) | Communication  (nonverbal) | Restrictive, repetitive, and stereotyped patterns of behavior | Abnormality of development evident at or before 36 month | |
| **Digit Span** |  |  |  |  |  |  |  |  |  |  |
| Digit Span, forward | -0.07 | 0.01 | 0.25 | -0.04 | -0.13 | -0.14 | -0.08 | 0.00 | -0.02 |  |
| Digit Span, backward | -0.03 | -0.12 | 0.01 | -0.15 | -0.15 | -0.15 | -0.11 | -0.09 | 0.08 |  |
| **Spatial Span** |  |  |  |  |  |  |  |  |  |  |
| Span Length | -0.19 | -0.17 | -0.15 | -0.25 | 0.02 | -0.15 | -0.14 | -0.27 | -0.02 |  |
| Total errors | 0.05 | 0.08 | -0.06 | -0.02 | 0.14 | 0.06 | -0.03 | -0.07 | 0.15 |  |
| **Spatial Working Memory** |  |  |  |  |  |  |  |  |  |  |
| Total errors | 0.15 | 0.16 | 0.01 | 0.03 | 0.00 | 0.26 | 0.17 | 0.16 | 0.15 |  |
| Strategy Utilization | 0.18 | -0.06 | -0.09 | -0.10 | -0.01 | 0.06 | 0.12 | -0.06 | -0.15 |  |
| **Stockings of Cambridge** |  |  |  |  |  |  |  |  |  |  |
| Problems solved in minimum moves | -0.14 | -0.22 | -0.20 | -0.12 | -0.01 | -0.29* | -0.25 | -0.23 | -0.22 |  |
| Total moves | 0.17 | 0.34* | 0.25 | 0.20 | 0.04 | 0.26 | 0.21 | 0.25 | 0.24 |  |
| **Intradimensional/Extradimensional shift** |  |  |  |  |  |  |  |  |  |  |
| Extra-dimensional shift errors | -0.04 | -0.05 | -0.16 | -0.02 | -0.01 | 0.14 | 0.09 | 0.01 | 0.09 |  |
| Pre-extra-dimensional shift errors | 0.26 | 0.27 | 0.19 | 0.08 | 0.18 | 0.18 | 0.16 | 0.12 | 0.08 |  |
| Completed stages | -0.15 | -0.11 | -0.02 | 0.06 | -0.19 | -0.24 | -0.26 | -0.08 | -0.13 |  |

*p<.05 **p<.01 ***p<.001

**Supplementary Table S7.** *The correlation between the performance of CANTAB and ADI-R: Age 13-18 (N=58)*

| Pearson Correlation Coefficients (r) | ADIR | | | | | | | | |
| --- | --- | --- | --- | --- | --- | --- | --- | --- | --- |
|  | Current | | | | Severe | | | | |
|  | Reciprocal social interaction | Communication  (verbal) | Communication  (nonverbal) | Restricted, repetitive, and stereotyped patterns of behavior | Reciprocal social interaction | Communication  (verbal) | Communication  (nonverbal) | Restrictive, repetitive, and stereotyped patterns of behavior | Abnormality of development evident at or before 36 month |
| **Digit Span** |  |  |  |  |  |  |  |  |  |
| Digit Span, forward | 0.30* | -0.79 | -0.76 | 0.09 | 0.15 | -0.09 | -0.01 | 0.01 | -0.19 |
| Digit Span, backward | 0.16 | 0.14 | 0.19 | 0.16 | -0.21 | -0.13 | -0.19 | -0.01 | -0.17 |
| **Spatial Span** |  |  |  |  |  |  |  |  |  |
| Span Length | 0.00 | 0.79 | 0.76 | -0.06 | 0.14 | 0.14 | 0.25 | -0.04 | 0.21 |
| Total errors | -0.22 | -0.98 | -0.99 | 0.27* | -0.10 | 0.00 | 0.03 | 0.23 | -0.06 |
| **Spatial Working Memory** |  |  |  |  |  |  |  |  |  |
| Total errors | 0.08 | 0.60 | 0.63 | 0.11 | -0.13 | -0.10 | -0.22 | 0.10 | -0.10 |
| Strategy Utilization | 0.13 | -0.99 | -0.98 | 0.12 | -0.15 | -0.10 | -0.24 | 0.10 | -0.12 |
| **Stockings of Cambridge** |  |  |  |  |  |  |  |  |  |
| Problems solved in minimum moves | 0.16 | 0.89 | 0.87 | 0.09 | 0.12 | 0.25 | 0.28* | 0.03 | 0.15 |
| Total moves | -0.17 | -0.94 | -0.93 | -0.16 | -0.09 | -0.26* | -0.27* | -0.06 | -0.17 |
| **Intradimensional/Extradimensional shift** |  |  |  |  |  |  |  |  |  |
| Extra-dimensional shift errors | 0.01 | -0.69 | -0.66 | -0.08 | -0.08 | -0.11 | -0.15 | -0.08 | -0.09 |
| Pre-extra-dimensional shift errors | 0.14 | -0.68 | -0.71 | 0.16 | 0.14 | 0.09 | 0.03 | 0.14 | -0.17 |
| Completed stages | -0.02 | 0.79 | 0.76 | -0.02 | -0.02 | -0.06 | -0.04 | -0.11 | -0.05 |

*p<.05 **p<.01 ***p<.001
