# Supplementary figures and images for: Deficits in executive functions among youths with autism spectrum disorders: an age-stratified analysis
Source: Psychol Med. 2016 Mar 21;46(8):1625–38. doi: 10.1017/S0033291715002238 (PMC4873936; doi:10.1017/S0033291715002238)

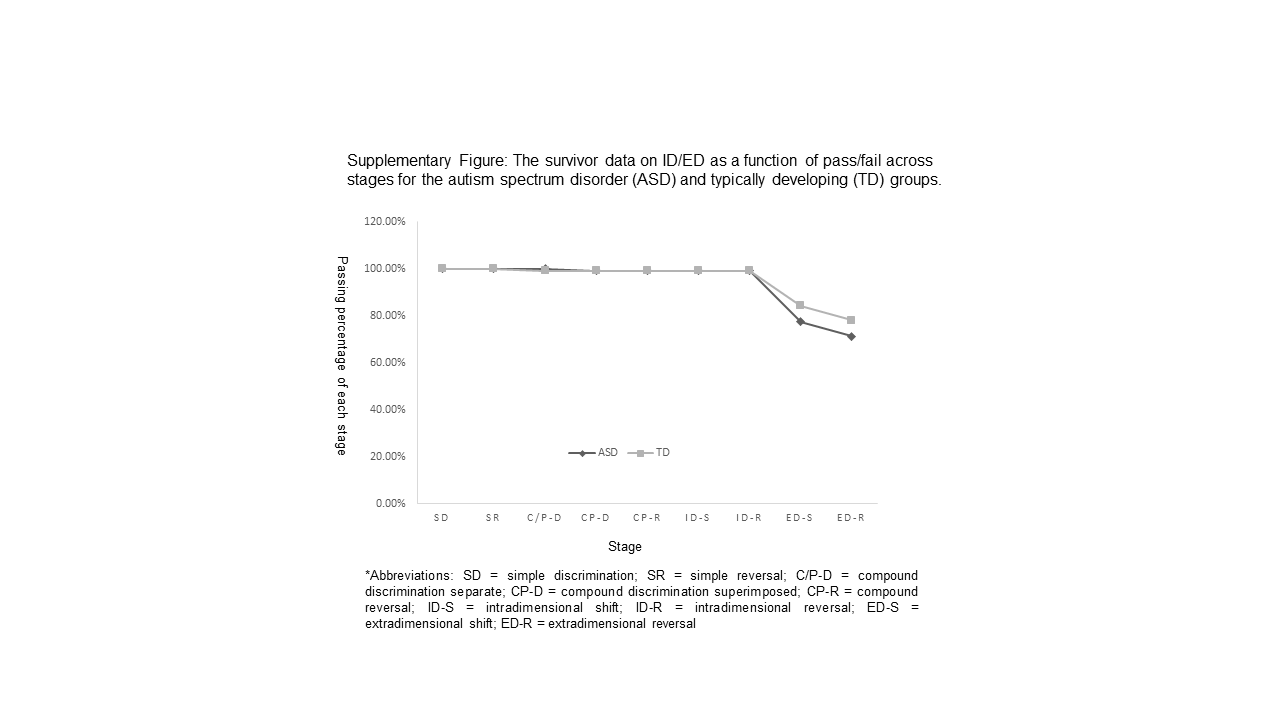

Supplement: Supplementary file 1 [file S0033291715002238sup.zip › S0033291715002238sup001.tif]
